# Assessment of the efficiency and effectiveness of health care systems in European Union countries before and during the COVID-19 pandemic

**DOI:** 10.3389/fpubh.2025.1592384

**Published:** 2025-07-08

**Authors:** Marek Biernacki

**Affiliations:** Department of Mathematics and Cybernetics, Wrocław University of Economics and Business, Wrocław, Poland

**Keywords:** excess death, COVID-19, effectiveness, treatment efficiency, healthcare systems

## Abstract

**Objectives:**

The aim of this study was to determine the extent to which the national healthcare systems of European Union countries used their medical potential in the fight against COVID-19. The analyzed period covered the years before the pandemic 2009–2018 and the COVID-19 pandemic. The analysis was conducted for all European Union’s countries. It concerned the evaluation not only of the effectiveness of treatment, but also of the efficiency, i.e., the use of resources in healthcare systems.

**Study design:**

Descriptive and analytical study on EUROSTAT data, Health Consumer Powerhouse, OECD Health Statistics, European Health Interview Survey (EHIS) and World Health Organization (WHO).

**Methodology:**

An index was proposed to measure the quality of healthcare systems’ activities, which simultaneously assesses the effectiveness and efficiency of treatment. The treatment results depend not only on management, but also on financial resources. EHCI, HLY, % of unmet medical needs were used to assess the effectiveness of treatment. Efficiency was calculated using with the DEA method based on total healthcare expenditure per capita at purchasing power parity. The final assessment is based on the difference in the index value from the pandemic period and before the COVID-19 pandemic.

**Results:**

The healthcare systems of small EU countries coped most effectively and efficiently during the COVID-19 pandemic: Denmark (0.6), Estonia (0.5), Austria (0.5), Slovenia (0.3), and the Czech Republic (0.3). However, large and non-rich EU countries fared much worse: Spain (−0.8), Hungary (−0.7) and Poland (−0.4). This group also unexpectedly included the Netherlands (−0.3)—a small, wealthy country that coped poorly with the challenges posed by the COVID-19 pandemic.

**Conclusion:**

Based on the analysis carried out in this paper, it can be concluded that the optimal (efficient and effective) use of medical resources did not depend only on the state of ownership and technological advancement of healthcare systems (!). The involvement of medical staff, society, and decisions of state authorities were equally significant.

## 1 Introduction

The Covid-19 pandemic marks the most significant global health crisis since the Spanish flu of 1918, a two-year ordeal that afflicted approximately 500 million individuals (1) and claimed the lives of over 50 million people (2). The Spanish flu notably affected young and healthy individuals aged 15 to 45, with a mortality rate slightly exceeding 10% (3). In contrast, over the course of a similar two-year period, COVID-19 infected more than 600 million individuals and resulted in over 6.5 million deaths. Notably, the mortality rate, representing the ratio of deaths to infections, stands at nine times lower than that of the Spanish flu. However, the majority of COVID-19 fatalities occurred among older individuals, aged over 65.^1^

In 1918, approximately (500/1650^2^)100% = 30% of the population was infected during the Spanish flu pandemic, whereas in 2020, the corresponding figure was approximately (600/7540^3^)100% = 8%, nearly four times smaller. Considering advancements in medicine, increased public awareness, and the lessons learned from the Spanish flu pandemic, the impact of the COVID-19 pandemic should have been significantly mitigated (4). In pursuit of public health objectives, countries have adopted varying strategies to combat Covid-19 amid pervasive uncertainty. Primary responses have ranged from pursuing herd immunity, exemplified initially by the UK, to strategies such as ‘flattening the curve’, a common approach adopted by most EU nations, and complete eradication, as seen in New Zealand. Some analysts argue that given the global ramifications of the disease, a centralised decision-making approach should have been adopted (5). According to the OECD, there was a significant surge in deaths during the pandemic’s peak in 2020–21 compared to the previous five years. Some countries hit hardest by excess mortality were Poland, the Czech Republic, Slovakia, Hungary, Mexico, Colombia, and the United States (6). This raises questions about how effectively these countries managed healthcare during the pandemic. Another indicator of healthcare performance that time was the percentage of unmet medical needs, with Hungary, Portugal, Latvia, Poland, and the United States leading the pack (7), all national healthcare systems were managed inefficiently and ineffectively. It’s essential to recall the Council of the European Union’s resolution 2011/C, which “emphasises the fundamental importance of the effectiveness of investments in future health systems, which should be measured and monitored by the relevant Member States,” while “a high level of quality of human health protection should be ensured while maintaining the principles: universality, accessibility, justice and solidarity.”^4^ The issue of inadequate monitoring of infection rates, mortality, and treatment outcomes extends beyond Europe to encompass the entire globe (8).

In the literature on the subject (17), there are works devoted to the analysis of the efficiency of treatment in European countries during the COVID-19 pandemic using the DEA method, e.g., in (9, 10), or the descriptive statistics and correlation and regression analysis method (11). Almeida analyzing 173 regions of Europe, showed, among other things, that regions with higher values of the health system efficiency index in 2017 had significantly higher rates of COVID-19 deaths in 2020 and 2021, suggesting the existence of a trade-off between health system efficiency and health system resilience during the COVID-19 pandemic. In poorer regions or countries, the high efficiency of health systems is a consequence of their underfunding. Similar conclusions were drawn by Coccia and Benati (12). Analyzing European countries, they showed that a lower COVID-19 mortality rate was strongly correlated with higher per capita healthcare expenditures, as well as a higher number of medical personnel and intensive care beds per 1,000 inhabitants. Therefore, underfunded regional health systems had a more difficult task of curing patients during the pandemic. Therefore, underfunded regional health systems faced greater challenges in treating patients during the pandemic. This naturally led to the question of optimal management of national healthcare systems during the pandemic in European countries.

Previous analyses of national healthcare systems in Europe and worldwide have focused either on the effectiveness or the efficiency of national or regional healthcare systems. As a result, a combined quantitative measure of both treatment effectiveness and the efficiency of resource utilization in healthcare systems has been proposed. To provide a complete picture, the management of national healthcare systems was compared before and during the COVID-19 pandemic.

The next study will examine the effectiveness and efficiency of national healthcare systems after the COVID-19 pandemic, in comparison to the periods before and during the pandemic. Simply put: which countries have “learned the lesson of the pandemic”?

## 2 Methodology

The aim of this article is to assess the performance of national healthcare systems, understood as the optimal use of physical and human resources during the COVID-19 pandemic. The analysis takes into account two key aspects: the economic aspect, that is system efficiency, and the professional aspect, that is treatment effectiveness. From the perspective of healthcare system management, this category concerns the rationalization of expenditures and the setting of reasonable goals — in this context, the improvement of the overall health of the population.

The efficiency of the healthcare system was calculated using the relative non-parametric DEA (Data Envelopment Analysis) method. The input variable was total healthcare expenditure per capita, expressed in purchasing power standards (PPS). As for the output variables, in the pre-pandemic period, the analysis used two synthetic indicators: the European Consumer Health Index (EHCI), which evaluates national healthcare systems from the patient’s perspective, and Healthy Life Years (HLY), a measure assessing the health status of a country’s population. Data on total healthcare costs and HLY were obtained from EUROSTAT, while EHCI values were sourced from Health Consumer Powerhouse.^5^ The pre-pandemic analysis covers the years 2009–2018.

During the pandemic, the input remained the same — total healthcare expenditures — while the output variables changed to reflect the new context. These included the percentage of medical needs met, as an indicator of how well the healthcare system responded to patient needs, and the excess mortality gap, as a synthetic measure of treatment effectiveness during the pandemic.

**Figure 1:**
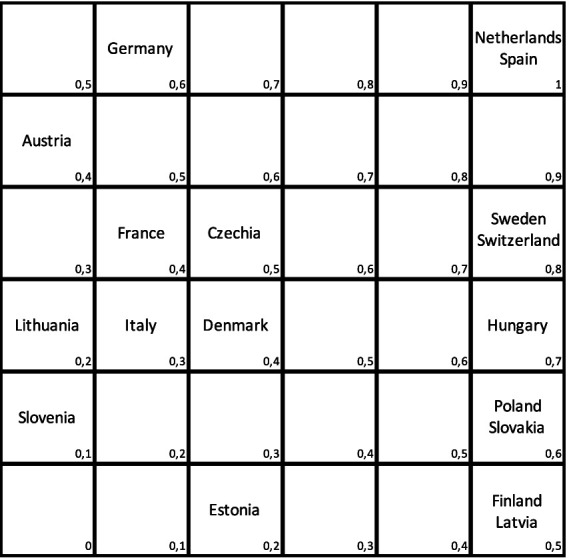
Effectiveness vs. efficiency, 2018. Source: Own study based on Eurostat data using deaR.

**Figure 2:**
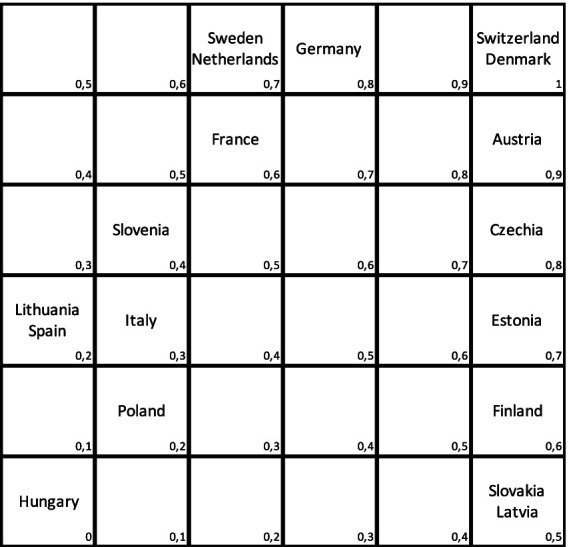
Effectiveness vs. efficiency, during pandemic. Source: Own study based on Eurostat using deaR.

The objective is to analyze the performance of national healthcare systems. For this purpose, a modified Pareto order was used to ensure that all units could be compared, i.e., the preference relation was consistent. Since the Pareto order is not linear, a modification was proposed. The results for efficiency and treatment effectiveness were normalized to the interval I = [0, 1]. This interval was then divided into six equal segments. A function with values in the range [0, 1] was defined on the square I^2^ (see Figures 1, 2). For a more comprehensive analysis, the optimal use of resources in healthcare systems, as well as their activities before and during the COVID-19 pandemic, were compared (see Table 1).

**Table 1:** Difference in the efficiency and effectiveness of national healthcare systems.

Country	Quality difference (effectiveness and efficiency) (during-before) pandemic
Czech Republic	0.3
Estonia	0.5
Lithuania	0
Slovakia	−0.1
Hungary	−0.7
Poland	−0.4
Latvia	0
Slovenia	0.3
Denmark	0.6
Finland	0.1
Sweden	−0.1
Spain	−0.8
Germany	0.2
France	0.2
Italy	0
Austria	0.5
Netherlands	−0.3
Switzerland	0.2

Source: Own compilation based on Eurostat data.

## 3 Before COVID-19

To assess the effectiveness of national healthcare systems before and during the COVID-19 pandemic, the percentage of unmet medical needs was used as a destimulant, based on the results of the European Health Interview Survey (EHIS).The relative, non-parametric DEA (Data Envelopment Analysis) method was employed to evaluate the efficiency of national healthcare systems. In DEA, decision-making units are defined with equal inputs and outputs. These units, primarily focused on specific public functions, are typically not profit-oriented. The efficiency of management with defined resources is assessed without relying solely on financial coefficients, making DEA widely applicable in studying public sector units (13). In maximising public health, this method employs output orientation and considers variable scale effects. Total expenditure on healthcare per capita serves as the input of the process, while the output comprises values such as EHCI (European Consumer Health Index) and HLY (Healthy Life Years). EHCI, the primary index reflecting healthcare quality in a country, assesses the efficiency of healthcare service provision from a patient’s perspective. This assessment encompasses five categories: 1. Patient rights and availability of information; 2. Waiting time for treatment; 3. Treatment outcomes; 4. Range and extent of services provided; 5. Pharmaceuticals.^6^

Based on the EHCI values from 2018, it can be observed that the best outcomes are achieved by the Nordic countries, along with Switzerland and Luxembourg. The Czech Republic and Slovakia are in the middle range, while Romania, Hungary, Poland and Bulgaria are at the lower end of the continuum among European countries.

The second result product utilised in the DEA method is HLY. Presumably associated with healthcare quality are both the duration of life and the duration of life in good health. While not directly linked, longevity and quality of health are influenced by various factors such as air pollution, cultural habits (e.g., alcohol consumption), natural disasters, among others. However, a significant level of dependency can be expected between healthcare quality and these factors. It is widely recognized that certain factors strongly influence life expectancy, such as reducing infant mortality rates and eradicating certain infectious diseases, which are achievements of modern medicine. Therefore, while not directly causative, healthcare quality does play a substantial role in overall life expectancy and quality of life.

The input for the DEA method is the total expenditure on healthcare in purchasing power standards per inhabitant (Eurostat, 2018).^7^ It is noteworthy to observe the wide range of countries’ expenditures. For instance, Switzerland allocated five times more per capita on healthcare compared to Bosnia and Herzegovina. Poland’s healthcare expenditure per capita falls below half of the EU average, representing approximately 6.3% of GDP, slightly higher than the EU average of 10%.

Correlations between inputs and outputs reveal that EHCI scores were above 0.8, while correlations between inputs and HLY hovered around 0.4. Thus, meeting the fundamental requirement for utilizing the DEA method, positive correlation, was established. The outcomes of calculations conducted with deaR are presented in Table 2. Among the studied countries, Sweden, Netherlands, and Poland emerge as the most efficient in the long term. Calculated efficiency in the Farrell sense indicates the potential percentage improvement in output volume given the then-existing expenditure levels. For example, Slovakia could have enhanced healthcare quality by 15.8% in 2009, by 5.8% in 2012, and by 14.9% in 2015, with 2018 identified as the optimal year for Slovakia. The Czech Republic performed well overall, except in the last year, where it achieved only a 1% improvement in output size with its inputs.

**Table 2:** Efficiency of national health systems measured by DEA.

Country	Efficiency score in 2009	Efficiency score in 2012	Efficiency score in 2015	Efficiency score in 2018
Czech Republic	1	1	1	1.01
Estonia	1	1	1	1.012
Lithuania	1.026	1.026	1.03	1.081
Slovakia	1.158	1.058	1.149	1
Hungary	1.057	1.057	1.055	1
Poland	1	1	1	1
Latvia	1.113	1.113	1	1
Slovenia	1.051	1.051	1.093	1.106
Denmark	1.056	1.016	1.081	1.006
Finland	1.071	1.071	1.003	1
Sweden	1	1	1	1
Spain	1.073	1.073	1.039	1
Germany	1.068	1.068	1.009	1.053
France	1.040	1.04	1.054	1.036
Italy	1.091	1.091	1.08	1.019
Austria	1.052	1.052	1.109	1.086
Netherlands	1	1	1	1
Switzerland	1.027	1.027	1.014	1

Source: Eurostat, Calculations with deaR.

As efficiency and outcomes are independent factors, we can devise a modified Pareto order (14). This involves positioning countries within a square grid, with efficiency measured by DEA on the horizontal axis and effectiveness measured by the percentage of unmet needs on the vertical axis. The further up and to the right a country is placed, the better its performance, while positioning towards the left and down indicates poorer performance (see Figure 1). In 2018, the healthcare systems of the Netherlands and Spain demonstrated the highest performance. Conversely, Germany and Austria had healthcare systems that were deemed “over-invested,” while Poland, Slovakia, Finland, and Latvia were characterised by under-financed healthcare systems. Slovenia exhibited the least efficient healthcare system.

To standardise the efficiency index within the range of [0, 1], countries lying on the square’s diagonal were assigned a value of 0.5. Subsequently, countries located immediately above the diagonal were assigned values incrementally increasing from 0.5 by 0.1, while those below the diagonal were assigned values decreasing from 0.5 by 0.1 (refer to Figure 1).

## 4 During COVID-19

During the pandemic, the European Foundation for the Improvement of Living and Working Conditions conducted an online survey on living conditions and quality of life. The conclusions drawn were as follows: The percentages reported were similar across Member States. During this period, healthcare institutions across Europe canceled or postponed services for non-COVID patients to manage the overwhelming influx of Coronavirus-infected patients requiring urgent care. For 85% of respondents, the unavailability of appointments or treatments was attributed to the pandemic. Additionally, 43% reported experiencing excessively long waiting times, while 37% refrained from seeking medical care out of fear of contracting COVID-19.^8^ Just as before the pandemic, the assessment of the effectiveness of national healthcare systems’ actions during the COVID-19 pandemic relied on the percentage of unmet medical needs. Based the website https://www.eurofound.europa.eu/en/publications/2020/living-working-and-covid-19 it is possibile to analyze the percentage of unmet medical needs during the initial 12 months of the pandemic. Arguably, the most comprehensive measure of the COVID situation in a given country is the rate of excess mortality deaths from all causes compared to projections based on the previous five years, divided by these projections. This metric effectively quantifies the fraction of deaths attributable to the pandemic. These deaths may not necessarily be attributed to COVID-19 specifically, but they rather represent fatalities that would not have occurred if the pandemic had not been present in the country. As in the pre-pandemic period, the efficiency of national health systems was assessed using the DEA method, with outputs including the percentage of medical needs met and the excess mortality gap. It is noteworthy that Healthy Life Years (HLY) during and after the pandemic may not be a reliable measure of treatment effectiveness (15). Unfortunately, there is a lack of European Health Consumer Index (EHCI) results from the COVID-19 pandemic and its aftermath. Table 3 presents the DEA analysis conducted for data during the pandemic. Correlations between expenditures and outcomes exceeded 0.63, thus meeting the condition for the applicability of the DEA method. The figures in the columns represent what could be achieved with the given resources, while the actual data are provided in parentheses. This allows us to ascertain what could have been achieved if the resources had been utilised more effectively. For instance, 950 (1800–850) deaths per million in Poland could have been prevented, whereas Czechia and Slovakia effectively utilised their resources.

**Table 3:** Efficiency of national health systems measured by DEA method and Target output.

Country	Efficiency score COVID-19	% of medical needs met	The excess mortality gap
Czech Republic	1	84 (84)	960 (960)
Estonia	1	81 (81)	3,050 (3050)
Lithuania	1.088	80 (74)	2,839 (2500)
Slovakia	1	77 (77)	1,350 (1350)
Hungary	1.167	75,9 (65)	2,335 (2000)
Poland	1.084	78 (72)	1800 (850)
Latvia	1	71 (71)	3,200 (3200)
Slovenia	1.087	82,6 (76)	2,228 (2050)
Denmark	1	90 (90)	4,250 (4250)
Finland	1	80 (80)	4,000 (4000)
Sweden	1.071	90 (84)	4,250 (3925)
Spain	1.093	81,9 (75)	2,786 (2550)
Germany	1.046	90 (86)	4,250 (3500)
France	1.047	87,9 (84)	3,245 (3100)
Italy	1.072	82,5 (77)	2,465 (2300)
Austria	1	88 (88)	3,150 (3150)
Netherlands	1.059	90 (85)	4,250 (3025)
Switzerland	1	90 (90)	3,350 (3350)

Source: Eurostat, Calculations with deaR.

Furthermore, we can construct a Pareto order for this purpose. To achieve this, we determine appropriate percentiles for both effectiveness (measured by DEA) and efficiency (measured by the percentage of unmet needs) (see Figure 2).

Once again, the countries positioned higher and to the right demonstrate better performance, while those situated lower and to the left indicate poorer performance. By comparing the positions of countries before and during the pandemic, Figures 1 and 2 reveal that only some remained stable, including Lithuania, Italy, and Latvia. For instance, Slovakia remained relatively stable, experiencing only a slight downward shift. Czechia moved to the right, indicating an improvement in relative effectiveness while maintaining stable results. In contrast, Poland’s effectiveness significantly declined, depicting a less favourable performance. Notably, Poland’s performance appears particularly poor, while Czechia has demonstrated commendable performance. Slovakia, while displaying high effectiveness, may have been constrained by limited resources, hindering its ability to achieve better results. During the COVID-19 pandemic, the most efficient national healthcare systems were observed in Switzerland and Denmark, with an efficiency index value of 1, followed closely by Austria (0.9), and Germany and Czechia (0.8). Conversely, the countries that struggled the most during the pandemic included Hungary (0), Lithuania, Spain, and Poland (0.2), with Italy (0.3) also experiencing challenges, despite maintaining its position from before the pandemic.

In summary, Table 1 highlights the disparities in the efficiency and effectiveness of national healthcare systems during and before the pandemic. Positive values indicate improvements, while negative values suggest a deterioration in the management of medical resources during the COVID-19 pandemic. Among EU countries, those whose healthcare systems have inefficiently utilised their medical potential during the COVID-19 pandemic include Spain, Hungary, Poland, and the Netherlands. Conversely, countries that have made the best use of their medical resources include Denmark, Estonia, Austria, Czechia, and Slovenia.

Latvia, which exhibited increasing economies of scale before the pandemic, did not enhance the efficiency of its healthcare system, likely due to underfunding. Countries with constant returns to scale before the pandemic experienced a decline in efficiency during the pandemic, with Slovakia showing a slight decline and Hungary experiencing a significant decrease (−0.7).

## 5 Discussion

The analysis of the quality of national healthcare system performance, including both treatment effectiveness and efficiency, before and during the COVID-19 pandemic is presented in Table 1. The health care systems of small EU countries coped most effectively and economically during the COVID-19 pandemic: Denmark (0.6), Estonia (0.5), Austria (0.5), Slovenia (0.3) and the Czech Republic (0.3). However, large and non-rich EU countries fared much worse: Spain (−0.8), Hungary (−0.7), Poland (−0.4). This group also unexpectedly included the Netherlands (−0.3)—a small, wealthy country that coped poorly with the challenges posed by the COVID-19 pandemic. It appears to have been a problem of systems management in crisis conditions, in particular the optimal use of hospitals and medical resources. Small countries could more easily implement vaccinations (Our World in Data, 2021)^9^ and non-pharmaceutical interventions (NPI), such as social distancing, quarantine, masks, etc. If we compare the total health expenditure per capita and the results of this analysis of European countries, then the analysis shows that the optimal (efficient and effective) use of medical resources did not depend only on the state of possession and technological advancement. Health care systems. Hence it can be concluded that the involvement of medical staff, society, and decisions of state authorities were significant. It seems that the issue primarily stemmed from systems management during crisis conditions, particularly in optimising the use of hospitals and medical resources. Small countries had an advantage in implementing vaccinations and non-pharmaceutical interventions (NPIs) like social distancing, quarantine measures, and mask mandates (16). The analysis highlights that achieving optimal (both efficient and effective) use of medical resources was not solely dependent on the state of possession and technological advancement of healthcare systems. Instead, the involvement of medical staff, societal cooperation, and decisions made by state authorities played significant roles in addressing the challenges posed by the pandemic.

## Data Availability

The original contributions presented in the study are included in the article/supplementary material, further inquiries can be directed to the corresponding author.

## Footnotes

^1^ https://ourworldindata.org/explorers/coronavirus-data-explorer

^2^World population during the Spanish flu pandemic.

^3^World population during COVID19 pandemic.

^4^ https://eur-lex.europa.eu/LexUriServ/LexUriServ.do?uri=OJ:C:2011:202:0010:0012:PL:PDF

^5^ https://healthpowerhouse.com/publications/

^6^ https://healthpowerhouse.com/publications/

^7^ https://ec.europa.eu/eurostat/databrowser/view/demo_r_mwk_05/default/table?lang=en

^8^ https://www.eurofound.europa.eu/en/blog/2021/protecting-access-healthcare-during-covid-19-and-beyond

^9^ https://ourworldindata.org/explorers/coronavirus-data-explorer
